# Continuous Theta Burst Stimulation Over the Right Orbitofrontal Cortex Impairs Conscious Olfactory Perception

**DOI:** 10.3389/fnins.2019.00555

**Published:** 2019-06-05

**Authors:** Gabriel Villafuerte, Adán Miguel-Puga, Oscar Arias-Carrión

**Affiliations:** ^1^Unidad de Trastornos del Movimiento y Sueño (TMS), Hospital General Dr. Manuel Gea González, Mexico City, Mexico; ^2^Plan de Estudios Combinados en Medicina (PECEM), Facultad de Medicina, Universidad Nacional Autónoma de México, Mexico City, Mexico; ^3^Centro de Innovación Médica Aplicada (CIMA), Hospital General Dr. Manuel Gea González, Mexico City, Mexico

**Keywords:** consciousness, continuous theta burst stimulation, olfaction, orbitofrontal cortex, perception, prefrontal cortex

## Abstract

The right orbitofrontal cortex (rOFC) has been proposed as the region where conscious olfactory perception arises; however, evidence supporting this hypothesis has all been collected from neuroimaging and lesion studies in which only correlation and not a temporal pattern can be established. Continuous theta burst stimulation (cTBS) causes a reversible disruption of cortical activity and has been used successfully to disrupt orbitofrontal activity. To overcome intrinsic limitations of current experimental research, a crossover, double-blind, prospective and longitudinal study was carried out in which cTBS was applied over the rOFC to evaluate its effect on odorant stimuli detection. All subjects received real and sham cTBS. Experimental procedures were done in two different sessions with a separation of at least one week between them to avoid carryover and learning effects. A total of 15 subjects completed the experiment, and their data were included in the final analysis (10 women, 5 men, mean age 22.40 ± 3.41). Every session consisted of two different measures of the conscious olfactory perception task: A baseline measure and one 5 min after cTBS/sham. Compared to baseline, marks in the olfactory task during the sham cTBS session increased (*p* = 0.010), while marks during the real cTBS session decreased (*p* = 0.017). Our results support the hypothesis that rOFC is an important node of a complex network required for conscious olfactory perception to arise. However, the exact mechanism that explains our results is unclear and could be explained by the disruption of other cognitive functions related to the rOFC.

## Introduction

Crick and Koch (1990) first proposed the neural correlates of consciousness as a means to explain conscious experiences. Neural theories of consciousness are mostly inferred from findings in the visual and auditory perceptual modalities and are not entirely applicable to other sensory modalities like olfaction (Stevenson and Attuquayefio, 2013). It is theorized that the endpoint integrationcortex of conscious olfactory perception is the three-layered piriform cortex (Merrick et al., 2014), which would set the neural correlate of olfactory consciousness in the paleocortex. Nevertheless, an alternative hypothesis focuses on the right orbitofrontal cortex (rOFC), a neocortical structure.

The correlation between rOFC activation and conscious olfactory perception was first described by Zatorre et al. (1992); thereafter, evidence from multiple neuroimaging studies (Kringelbach and Rolls, 2004; Gottfried and Zald, 2005; Seubert et al., 2013) and lesion studies have supported this relationship (Zatorre and Jones-Gotman, 1991; Li et al., 2010). However, a major drawback of using this type of studies to establish causality is the bias inherent to the different components of neuroimaging techniques and temporal resolution. Also, contradictory evidence exists regarding rOFC and conscious olfactory perception: direct cortical electrical stimulation via subdural electrodes over rOFC failed to produce positive olfactory perception in children with epilepsy (Kumar et al., 2012); and not all subjects with rOFC damage have olfactory impairment (Jones-Gotman and Zatorre, 1988).

Repetitive transcranial magnetic stimulation (rTMS) is a safe, non-invasive method to study cortical function. It is based on the Faraday’s Law of Induction that describes how a changing magnetic field creates an electrical current and conversely, how an electrical current running through a coil generates a magnetic field. Which, in the case of rTMS, goes through the skull and modifies the activity of the underlying neurons (Kobayashi and Pascual-Leone, 2003). By applying the magnetic pulses repetitively, it is possible to, transiently, disrupt cortical activity and therefore create an experimental approach in which a temporal pattern can be established.

Several studies have used rTMS to successfully impair the activity of the rOFC (Nauczyciel et al., 2014). Due to shorter stimulation time, continuous theta burst stimulation (cTBS) was shown to be the most efficient modality of rTMS for this purpose (Costa et al., 2011; Volman et al., 2011; Cerasa et al., 2015; Ryals et al., 2016; Hanlon et al., 2017). To our knowledge, none of these attempts to disrupt rOFC evaluated its effect on olfactory perception.

So that the sensory modality of olfaction can be integrated with the current paradigm of consciousness research, experimental evidence of the relationship between rOFC and conscious olfactory perception is needed. As cTBS causes a reversible disruption of cortical activity (Huang et al., 2005; Hanlon et al., 2017), we hypothesized that cTBS over rOFC would impair the ability of a subject to consciously detect odorant stimuli.

## Materials and Methods

### Participants

Subjects between 18 and 30 years old were recruited for the present study from January 2018 until October 2018. Subjects did not have a history of brain disorders, sensory perception dysfunction nor were consuming any drug with central nervous system activity. All subjects were screened for olfactory dysfunction prior to the study using the Quick Smell Identification Test^TM^ (Sensonics International, Haddon Heights, NJ). A total of 21 subjects were recruited and screened. Seventeen right-handed subjects satisfied the inclusion/exclusion criteria; however, two of these subjects did not finish the experiment. The data of the 15 subjects that completed the experiment are included in the final analysis (10 women, 5 men, mean age 22.40 ± 3.41). Subjects provided written informed consent to participate in the study. The study protocol was reviewed and accepted by the research and ethics committee of Hospital General “Dr. Manuel Gea González”; protocol number 49-54-2018 and was conducted per the Declaration of Helsinki.

### Experimental Design

All subjects received both real and sham cTBS. Experimental procedures were done in two different sessions with at least one week of separation to avoid carryover and learning effects. The order of stimulation was randomized to avoid possible bias. Both subjects and the researcher applying the olfactory task were blinded to the type of each session. The cTBS protocol was applied by a researcher that had no participation in the conscious olfactory perception task.

**FIGURE 1:**
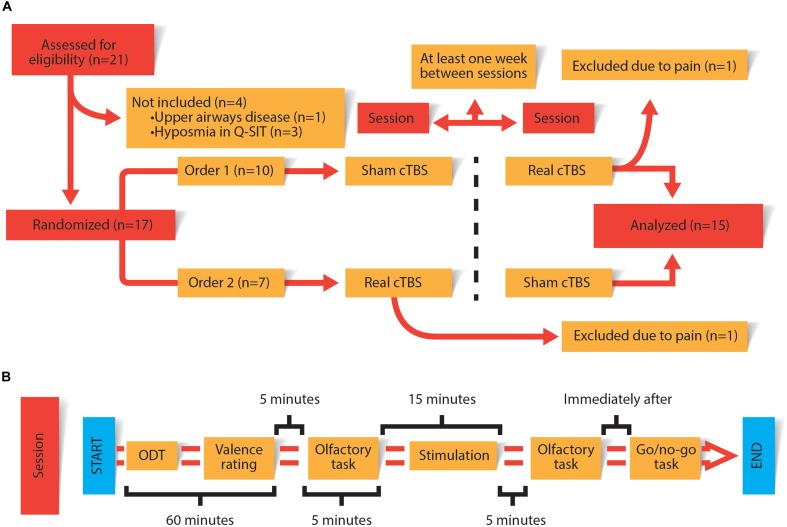
**(A)** General design of the experiment carried out on each of the subjects. **(B)** Procedures carried out in each session. The duration of each procedure is specified in the figure.

Every session consisted of collecting two different measures of the conscious olfactory perception task: at baseline and another 5 min after cTBS/sham. An olfactory detection threshold (ODT) task was realized for every odorant stimuli used in the conscious olfactory perception task. After establishing ODT, a pleasantness rating was asked for every odorant stimuli. Finally, as a positive control of the impairment of the rOFC a Go/no-go task was administered to the subject 10 min after CTBS/sham (Drummond et al., 2017). All procedures are summarized in Figure 1.

### Odorant Stimuli

To avoid possible bias in the experimental response of the subjects due to the pleasantness of the odorants (Bensafi et al., 2002), we chose odorants with three different values of pleasantness: Valeric acid (Sigma-Aldrich 240370) which has an unpleasant rotten-like odor, alpha-pinene (Sigma-Aldrich 147524) which has a neutral wood-like odor and amyl acetate (Sigma-Aldrich 109584) which has a pleasant rose-like odor. Two-fold serial dilutions were prepared for each of the three odorants ranging from 8% concentration (labeled number 1) to 2.5 × 10^-4^ % concentration. Propylene glycol (Sigma-Aldrich P4347) was used as the solvent in all dilutions.

### Olfactory Detection Threshold Task

Olfactory detection threshold is found by determining the smallest concentration in which a subject can detect an odorant. Due to the many variables that can alter this threshold from one day to another (Marioni et al., 2010; Ketterer et al., 2011; Sorokowska et al., 2015), ODT was calculated individually for each odorant at the beginning of each session. A forced choice, single staircase paradigm as described by Doty (2015) was used to establish the ODT. A custom made olfactometer, [design based on previously published papers (Lundström et al., 2010; Lowen et al., 2016)], was used to administer the odorant stimuli to both nostrils for 5 s. The concentration obtained at the ODT was then used in the conscious olfactory perception task.

### Odorant Valence Rating

Odorant valence is defined as the pleasantness of an odorant to an individual subject. As personal preferences influence the valence evoked by the odorants, at the start of every session we tested each subject’s valency of the odorants at the ODT concentration. A visual analog scale, as described by Clepce et al. (2014) was used. For each odorant, subjects were presented with a paper with a straight line of 20 cm. A mark and a zero (0) were printed at the center of the line; negative (“---”) and positive (“+ + +”) signs were printed at the left and right extremes of the line, respectively. Subjects were instructed to rate the odorants; the left extreme represented the most unpleasant odorant they could recall, and the right extreme represented the most enjoyable odorant they could recall. The zero indicated neither an enjoyable nor unpleasant experience, i.e., a neutral odor. The subject was instructed to mark the perceived odorant valence at the corresponding place along the line. Distance from the subject’s mark to zero was measured and used as a continuous variable of pleasantness.

### Conscious Olfactory Perception Task

**FIGURE 2:**
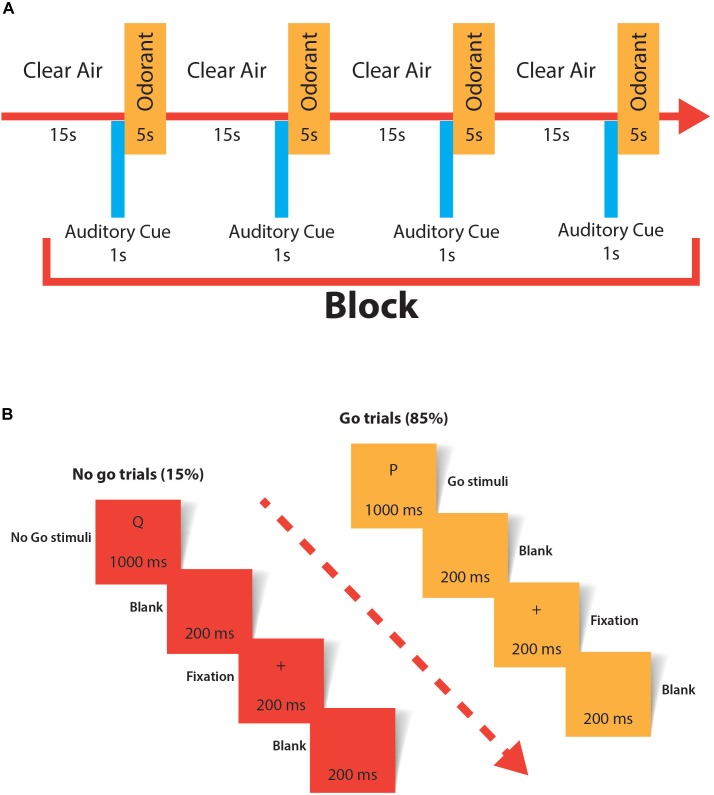
**(A)** The experimental paradigm used to evaluate conscious olfactory perception. All odorants were randomized to avoid order bias. One of the four stimuli was odorless (propylene glycol). The task consisted of two blocks of stimulation separated by 1 min. The task was applied twice, at baseline and after the real/sham cTBS stimulation. **(B)** Experimental paradigm used in the Go/no-go task. A total of 120 stimuli were applied (85% go trials and 15% no-go trials); the first 20 stimuli were considered training and not used for analysis.

Assessing conscious perception in any sensorial modality is controversial; however, it is widely accepted that self-reported experiences are a valid way to test consciousness (Dehaene and Changeux, 2011). We designed a similar task to one published by Li et al. (2010). The previously named odorants plus an odorless stimulus (propylene glycol) were presented directly to the subject’s nostrils using our custom made olfactometer. A block of stimulation consisted of the 4 stimuli administered one after another with a separation of 15 s of clean air between stimuli in a randomly sequenced order. The task consisted of two blocks of stimulation separated by 1 min. All subjects were blindfolded to prevent visual input interference. The subjects were instructed to push a button only if they detected an odorant. To focus the attention of the subject, they were instructed to sniff for odorant only after an auditory cue. Correct answers (true positives and true negatives) and mistakes (false positives and false negatives) were recorded. The conscious olfactory perception task is resumed in Figure 2A.

### Go/No-Go Task

The rOFC is widely associated with motor response inhibition (Rolls, 2004). Alteration of motor response inhibition has been reported in humans (Drummond et al., 2017) and non-human primates (Iversen and Mishkin, 1970) after disrupting activity in the orbitofrontal cortex. A Go/no-go task (Wessel, 2017) was used to measure motor response inhibition in our subjects. We used the Psychopy software (Peirce, 2007) to design and administer the Go/no-go task. Total marks and mistakes were recorded.

Signal detection theory was the approach we used to determine changes in the hit rate and correct rejection rate between trials. Hits (true positives), false alarms (false positives), misses (false negatives) and correct rejections (true negatives) were calculated by pooling the results of all the subjects in each of the trials (baseline sham cTBS, post-sham cTBS, baseline real cTBS and post-real cTBS). To simultaneously compare hit rates (hits/hits + misses) and correct rejection rates (correct rejections/correct rejections + false alarms) of different trials in a straightforward graphical approach, the method described by Newcombe (2001) was used. This method calculates the difference with 95% confidence intervals between the hit rate and correct rejection rate of two conditions. All the calculations of this method were performed using the Excel spreadsheets provided as downloadable content in a book by the same author (Newcombe, 2013). Parameter λ is the mixing parameter that represents the weighting of the hit rate and the correct rejection rate. Statistical significance was assumed when the 95% confidence intervals did not cross zero (Newcombe, 2001). Details of the Go/no-go task are described in Figure 2B.

### Continuous Theta Burst Stimulation (cTBS)

A cTBS protocol was used to disrupt the activity of the rOFC. We used a Magstim Rapid 2 machine (Magstim Co., Whitland Wales, United Kingdom) to deliver the magnetic pulses. A figure-of-eight coil with a 70 mm of diameter was used for all experimental procedures. The cTBS protocol implemented here was first described by Huang et al. (2005); it consists of 20-s trains of 3 pulses at 50 Hz separated by 200 ms, for a total of 300 pulses. Machine output intensity was set at 80% of the individual active motor threshold (AMT). AMT was calculated at each session for the left first dorsal interosseous. We defined AMT as the minimum machine output intensity needed to produce motor evoked potentials of at least 200 μV, while the muscle contraction was at 20% of the maximum contraction (Huang et al., 2005). The inhibition caused by cTBS lasts for at least 15 min after the stimulation (Huang et al., 2005).

To locate the rOFC we combined the 10–20 EEG coordinates for Fp2 and neuronavigation using the Visor2 software and Polaris Vicra 3d camera. The Fp2 coordinates were located in the skull of the subject as described by Herwig et al. (2003). The anatomical location was then digitalized and merged with a standardized MRI with the visor2 software. The real cTBS was applied tangentially to the stimulation site with the handle pointing upward, while the sham cTBS was applied in the same site but with the coil turned 90° away from the stimulation point. Subjects were blindfolded and asked to close their eyes during stimulation.

### Statistical Analysis

All statistical analyses were performed with an IBM SPSS statistics 20 package for Windows. Figures were created with GraphPad software for Windows. Differences between sham and real cTBS conditions were assessed with paired *T*-tests that compared ODT, odorant valences, AMT and the Go/no-go task results. A two-way repeated measures ANOVA was carried out to establish the interaction between time (baseline vs. post-stimulation) and type of stimulation (sham vs. real) in the marks obtained in the olfactory conscious detection task. A Spearman’s rank-order correlation test was used to determine if there was a relationship between mistakes in the Go/no-go task and mistakes made in the conscious olfactory task. Values of *p* < 0.05 were considered statistically significant unless otherwise specified.

## Results

**FIGURE 3:**
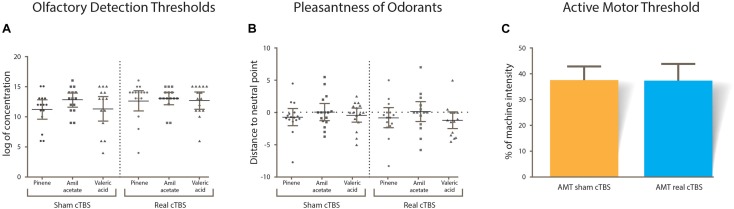
**(A)** Results from olfactory detection thresholds. **(B)** Pleasantness ratings for odorants; most of the subjects rated all three near zero (horizontal dotted line). **(C)** Active motor threshold between sham and real cTBS sessions. No statistical differences were found in any of the CTBS conditions.

A summary of the results is displayed in Table 1. In general, variables that could affect the outcome of the study (odorant valence, AMT, ODT) did not differ between session type (real or sham cTBS). All variables were normally distributed and therefore paired sample *T*-tests were used. There was no statistical difference in the ODTs of each odorant within sessions (alpha-pinene *t*(14) = -1.142, *p* = 0.273, amil acetate *t*(14) = -0.472, *p* = 0.644, valeric acid *t*(14) = -1.558, *p* = 0.142; Figure 3A). Odorant pleasantness was not statistically different between sessions and within odorants (alpha-pinene *t*(14) = 1.427, *p* = 0.176, amil acetate *t*(14) = -0.718, *p* = 0.484, valeric acid *t*(14) = -0.307, *p* = 0.487; Figure 3B). The AMT was not different between sessions *t*(14) = -0.307, *p* = 0.764; Figure 3C.

**Table 1:** Results of the different variables measured in both sessions.

Variables	Sham cTBS	Real cTBS	*p*-value
Pinene Threshold	11.20 ± 2.86	12.60 ± 3.02	0.273
Amil acetate Threshold	12.73 ± 2.09	13.00 ± 1.85	0.644
Valeric acid Threshold	11.27 ± 3.59	12.67 ± 2.55	0.142
Pinene Valency (mm)	-0.25 ± 2.54	-1.22 ± 2.54	0.176
Amil acetate Valency (mm)	0.17 ± 2.54	0.53 ± 2.54	0.484
Valeric acid Valency (mm)	-0.56 ± 2.07	-0.98 ± 2.38	0.487
Active Motor Threshold (%)	37.53 ± 5.94	37.93 ± 6.30	0.764
Olfactory Marks baseline	4.87 ± 1.06	5.53 ± 1.13	0.106
Olfactory Marks post-stimulation	5.60 ± 0.91	4.73 ± 1.16	0.022
Mistakes Go/no-go task	1.20 ± 1.37	2.07 ± 1.91	0.007


*All the displayed results are means and standard deviations. *p* values are calculated with paired *t*-test statistics.*

**FIGURE 4:**
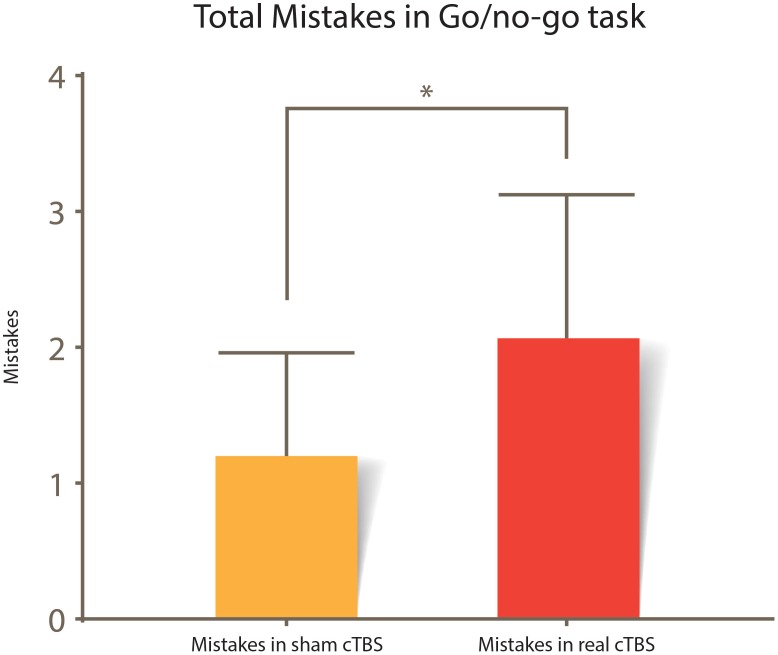
Total mistakes committed in the Go/no-go task. A statistical difference between sham vs. real cTBS conditions is noted *t*(14) = 3.166, *p* = 0.007. Error bars represent 95% CI.

A paired sample *T*-test was used to assess whether there was a statistically significant difference between the mistakes committed in the Go/no-go task in the real cTBS condition compared to the sham cTBS condition. There were no outliers in the data, as no data point was greater than 1.5 box lengths from the edge of the boxplots. The difference in mistakes for the real cTBS and the sham cTBS fulfilled the normality assumption, as assessed by Shapiro–Wilk’s test (*p* = 0.246) and normal Q–Q plots. Participants had more mistakes in the real cTBS condition (2.06 ± 1.91) compared with the sham cTBS condition (1.20 ± 1.37); this was interpreted as a statistically significant increase of 0.87 (95% CI, 0.28 to 1.45) mistakes in the real cTBS condition, *t*(14) = 3.166, *p* = 0.007 *d* = 0.82; Figure 4.

As the Go/no-go task was intended to reflect the function of the rOFC, we hypothesized that the number of mistakes in the Go/no-go task after the real cTBS test would correlate with the number of mistakes in the olfactory task after the real cTBS. A Spearman’s rank-order correlation was used to prove this hypothesis. No correlation was found between the variables *r*_s_ (13) = 0.142, *p* = 0.613.

**FIGURE 5:**
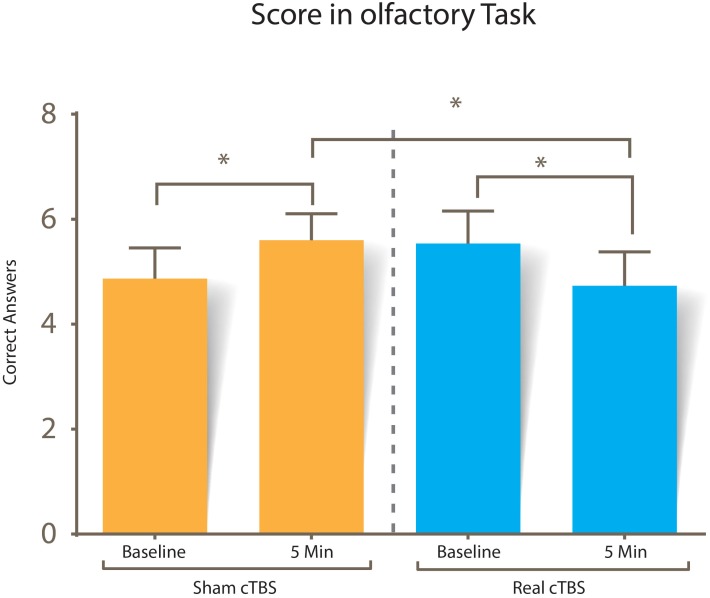
Score in the conscious olfactory perception task. No statistical significance was found when comparing the correct answers of both session types at baseline (*p* = 0.106), but there was statistical significance between sham vs. real cTBS stimulation scores (*p* = 0.022), and when baseline was compared with scores after both sham (*p* = 0.010) and real cTBS (*p* = 0.017).

Scores of the olfactory task in each of the different conditions are displayed in Figure 5. A two-way repeated measures ANOVA was performed to determine the effect of cTBS between baseline and post-stimulation in the rOFC. Using standardized residuals, no outliers were found in our data (values ± 3 were considered outliers). Scores in the olfactory consciousness task were distributed normally (*p* > 0.05) except at the pre-cTBS trial (*p* = 0.018), as assessed by Shapiro–Wilk’s test of normality on the standardized residuals; however, Q–Q plots and box plots showed symmetry in the non-normal data, so no further adjustment was made to the data.

A statistically significant two-way interaction between type of cTBS (sham vs. real) and time (baseline vs. post-stimulation) was found in the number of correct answers in the olfactory consciousness task, *F*(1, 14) = 25.020, *p* < 0.0005 partial η^2^ = 0.641. No statistically significant main effects were observed for time and type of cTBS.

The interaction was further analyzed using paired sample *T*-tests. Given that there were multiple tests for simple main effects, the criterion for significance was adjusted to 0.025. Baseline correct answers were not statistically different between sham (mean = 4.86 SD = ±1.06) and real cTBS sessions (5.56 ± 1.12) *t*(14) = -1.726, *p* = 0.106. This changed after the intervention, since correct answers after the sham cTBS intervention (mean = 5.60 SD = ±0.91) were statistically different than correct answers after the real cTBS intervention (4.73 ± 1.16), *t*(14) = 2.578, *p* = 0.022, *d* = 0.665.

Time affected performance in both the real and sham cTBS session; but, in opposite directions: correct answers pre-intervention (mean = 4.86 SD = ±1.06) were statistically different than post-intervention (mean = 5.60 SD = ±0.91) in the sham session *t*(14) = -2.955, *p* = 0.010, *d* = -0.762, while correct answers in the real cTBS pre-intervention (5.56 ± 1.12) were also statistically different than those after the intervention (4.73 ± 1.16) *t*(14) = -2.703, *p* = 0.017, *d* = 0.697.

**FIGURE 6:**
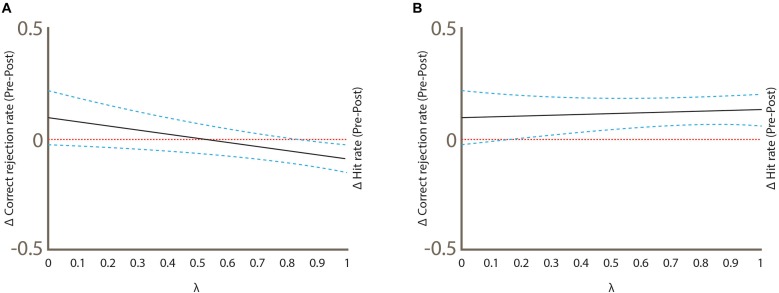
Graphical analysis of hit rate and correct rejection rate between test conditions. Statistical significance was achieved when the CI 95% did not cross zero (the horizontal pointed line). **(A)** Sham condition **(B)** Real condition. Note that the delta hit rate changes between conditions.

Figure 6 shows the graphical analysis of hit rate and correct rejection rate between conditions. The difference between baseline vs. post-sham hit rate was statistically significant (Δ = -0.0889, 95% CI, -0.151 to -0.026; Figure 6A), while the baseline vs. post-sham correct rejection rate was not (Δ = 0.100, 95% CI, -0.025 to 0.221; Figure 6A). In the baseline vs. post-real cTBS hit rate difference was also significant but in opposite direction (Δ = 0.133, 95% CI, 0.0594 to 0.205; Figure 6B), while the correct rejection rate was again not significant (Δ = 0.100, 95% CI, -0.024 to 0.2187; Figure 6B).

## Discussion

Elucidating the function of the rOFC in conscious perception is not an easy task. Several arguments suggest that the rOFC is the neural substrate for conscious olfactory perception. First, the rOFC is a neocortical structure; all of the other sensorial modalities in which neural correlates of consciousness have been closely mapped to brain processes are located in neocortical structures (Parvizi et al., 2012). Second, the rOFC has a functional connection to the thalamus (Plailly et al., 2008) and this structure is considered to be essential for the rise of conscious experience (Edelman and Gally, 2013; Nigri et al., 2016). Third, the rOFC integrates multimodal experiences (Rolls and Baylis, 1994) and conscious experiences require multimodal integration to arise (Mori et al., 2013); therefore, the rOFC may be fulfilling this role for the olfactory modality. And lastly, previous neuroimaging and lesion studies showed that rOFC activity correlates better with conscious perception than any other olfactory structure (Poellinger et al., 2001).

Previously, Li et al. (2010) reported a subject with a specific post-stroke lesion of the rOFC and consequent loss of conscious olfactory perception. As the integrity of the rest of neural olfactory areas was ensured by neuroimaging techniques and preservation of unconscious olfactory perception was corroborated, this study gave major support to the hypothesis of the rOFC as the neural correlate of olfactory consciousness. However, it is impossible to determine if the change in the neural-function is a direct consequence of the lesion or to the lesion-related plasticity (Rorden and Karnath, 2004).

Our results support the hypothesis that the rOFC is an important node of the complex network required for conscious olfactory perception. As measured by our task, cTBS over rOFC impaired conscious olfactory perception affecting a statistically significant interaction between time and type of stimulation, but without statistically altering the main effects. This can be interpreted as no overall effect of either time or type of stimulation but to evidence of a crossover interaction. The effect of time on the score was opposite depending on the stimulus received. Our results showed the score improved in respect to baseline after sham cTBS, while the contrary pattern was observed in the real cTBS. This result is corroborated by signal detection theory: the hit rate increased after sham cTBS compared with baseline, while it decreased after real cTBS. The hit rate is a proportion between the hits (true positives) and the number of odorant stimuli presented in each session; this means that cTBS affected the number of hits subjects scored. Correct rejection rates were not affected in both sham and real cTBS.

Score improvement after sham cTBS could be attributed to a learning process or an improved sensitivity to the odors; repeated exposure to odorants may cause experience-dependent plasticity in the piriform and orbitofrontal cortex (Li et al., 2006). A study by Huang et al. (2007) found cTBS generates a long-term depression-like activity in the brain which may impair the normal plasticity of rOFC. Therefore, according to these studies, the decrease in scores may be due to a failure in the learning process, and not necessarily to a failure in conscious perception. Nevertheless, the study of Li et al. (2006) used a much higher concentration of odorants, and the task reported assessed the similarity of odorants, not the presence or absence of them. If the effects observed in our experiments were due to an interruption in the learning process, not only the right OFC but also the piriform cortex and the left OFC, would have to have been affected, which is unlikely due to the high focality of cTBS.

Attention to stimuli strengthens the connection of the mediodorsal nucleus of the thalamus with the OFC (Plailly et al., 2008). Disruption of the rOFC activity may generate a malfunction of the mediodorsal thalamus-OFC circuit and therefore impair performance of the task. To overcome this limitation, subjects were always cued with an auditory stimulus before application of the odorant; also, it is unlikely that diminished attention would affect our results given that conscious olfactory perception may arise with a minimum of attention (Carskadon and Herz, 2004).

As low odorant concentration could diminish conscious olfactory perception due to under stimulation of the system and a high odorant concentration could produce habituation to the stimuli (Merrick et al., 2014), we individualized ODT for each of the odorants in every session. Overall, the ODT of our subjects did not change between sessions; therefore, the probability of bias due to this factor is reduced. Although, the role of the rOFC in habituation is more complex than it may seem. Poellinger et al. (2001) showed that the rOFC might top-down habituation of the system; therefore, the possibility that cTBS caused habituation to stimuli by inhibiting rOFC cannot be discarded. However, Poellinger used long-lasting stimuli (at least 60 s), whereas our stimuli lasted 5 s. Therefore, it is unlikely that the mechanism of impaired performance is due to facilitated habituation.

Emotionally salient odorants affect high order olfaction functions in the rOFC (Gottfried et al., 2002). To prevent a possible bias related to the personal and emotional response that odorants evoked in the subjects, three different odorants with different pleasantness were chosen. Unexpectedly, most of the subjects rated the pleasantness of odorants near zero (a neutral odorant, neither enjoyable nor unpleasant) and no differences between sessions were noted. This can be explained by the low concentration in which the odorants were administered, considering that low stimuli concentration evoke low hedonic responses (Doty, 1975).

Disruption of the rOFC in non-human primates (Iversen and Mishkin, 1970) and humans, impairs performance in the Go/no-go task (Drummond et al., 2017). To ensure an adequate effect of the cTBS, we used the Go/no-go task performance as a proxy measurement of correct cTBS application. Subjects had more mistakes in the real cTBS session compared with the sham cTBS session, which indicates subjects presented motor disinhibition in the real cTBS session; this result is consistent with the current literature (Drummond et al., 2017). No visual changes were reported during or after the stimulation, which in addition to the results of the Go/no-go task reveal silence in the visual sensory modality. The rOFC has been linked to decision making when exposed to ambiguous olfactory stimuli (Bowman et al., 2012). Then, both motor disinhibition and an impaired ability to recognize olfactory stimuli presented in a random sequence could explain our results; however, new experiments are needed to confirm this hypothesis.

The other limitations of our study are: As neuroimaging was unavailable to corroborate the functional inhibition of rOFC, a Go/no-go task was used to resolve this limitation. Future studies employing an appropriate neuroimaging technique are necessary to corroborate these findings further. We could not test for the implication of brain lateralization on the studied phenomenon; a common bias could explain the existing paradigm that implicates the rOFC and not the left OFC in conscious olfactory perception: most of the studies only include right-handed subjects. More studies exploring changes between left vs. right cTBS, uni- vs. bilateral odorant presentation and left- vs. right-handed are needed to clarify brain lateralization in the function of OFC in conscious olfactory perception. Another limitation that should be remarked is genre: two-thirds of our subjects were women; this could implicate a bias and an obstacle for external validity. Women have a different sensitivity for olfaction, and their olfaction can be altered by the menstrual cycle and contraceptive pills; these variables were not controlled in the present study (Navarrete-Palacios et al., 2003; Derntl et al., 2013). Bigger sample sizes are needed to accurately describe whether gender affects the role of the rOFC in conscious olfactory perception.

## Conclusion

Previous research with neuroimaging and lesion studies has shown that rOFC activity may be deeply correlated with conscious olfactory perception. We confirmed this relationship and also showed that a temporal pattern between rOFC and conscious olfactory perception could be drawn. However, our results should be read carefully; as stated before, there are many higher-order olfactory functions attributed to the OFC. More experiments that are specifically designed to test learning, attention, habituation and multimodal integration of olfactory odorants are needed to correctly dissect how the cTBS affects the wide spectrum of olfactory functions that are attributed to the rOFC.

cTBS over the rOFC appeared to affect conscious olfactory perception. The exact mechanism of this impairment is unclear and could be explained by the disruption of other cognitive functions related to the rOFC; however, the continued application of cTBS in research related to the neuroscience of olfaction could help to answer many of these questions.

## Ethics Statement

The study protocol was reviewed and accepted by the research and ethics committee of Hospital General “Dr. Manuel Gea González”; protocol number 49-54-2018 and was conducted per the Declaration of Helsinki.

## Author Contributions

GV and AM-P were responsible of performing the experiments. All authors read and approved the final version of this manuscript and contributed to the data analysis and experimental design.

## Conflict of Interest Statement

The authors declare that the research was conducted in the absence of any commercial or financial relationships that could be construed as a potential conflict of interest.
